# Phosphorylation and sulfation share a common biosynthetic pathway, but extend biochemical and evolutionary diversity of biological macromolecules in distinct ways

**DOI:** 10.1098/rsif.2022.0391

**Published:** 2022-08-03

**Authors:** M. A. Lima, T. R. Rudd, D. G. Fernig, E. A. Yates

**Affiliations:** ^1^ Centre for Glycosciences, Keele University, Keele ST5 5BG, UK; ^2^ School of Life Sciences, Keele University, Keele ST5 5BG, UK; ^3^ Analytical and Biological Science Department, National Institute of Biological Standards and Control (NIBSC), Blanche Lane, South Mimms, Potters Bar EN6 3QG, UK; ^4^ Department of Biochemistry and Systems Biology, ISMIB, University of Liverpool, Liverpool L69 7ZB, UK

**Keywords:** phosphorylation, sulfation, macromolecules

## Abstract

Phosphate and sulfate groups are integral to energy metabolism and introduce negative charges into biological macromolecules. One purpose of such modifications is to elicit precise binding/activation of protein partners. The physico-chemical properties of the two groups, while superficially similar, differ in one important respect—the valency of the central (phosphorus or sulfur) atom. This dictates the distinct properties of their respective esters, di-esters and hence their charges, interactions with metal ions and their solubility. These, in turn, determine the contrasting roles for which each group has evolved in biological systems. Biosynthetic links exist between the two modifications; the sulfate donor 3′-phosphoadenosine-5′-phosphosulfate being formed from adenosine triphosphate (ATP) and adenosine phosphosulfate, while the latter is generated from sulfate anions and ATP. Furthermore, phosphorylation, by a xylosyl kinase (Fam20B, glycosaminoglycan xylosylkinase) of the xylose residue of the tetrasaccharide linker region that connects nascent glycosaminoglycan (GAG) chains to their parent proteoglycans, substantially accelerates their biosynthesis. Following observations that GAG chains can enter the cell nucleus, it is hypothesized that sulfated GAGs could influence events in the nucleus, which would complete a feedback loop uniting the complementary anionic modifications of phosphorylation and sulfation through complex, inter-connected signalling networks and warrants further exploration.

## 1. Introduction

The evolution of complex organisms and the need to coordinate precise responses to changing conditions selected molecular systems able to recognize and bind partners to initiate, regulate or terminate the appropriate biochemical processes. Macromolecular interactions that are coordinated by charge–charge interactions, combined with conformational compatibility and augmented by weaker, but numerous, hydrogen bonding and other interactions, collectively provide sufficient affinity, selectivity and specificity to fulfil this role. Biochemical processes have evolved to rely heavily, although not exclusively, on phosphorylation to add the charged moieties, while phosphatases remove them. Such modifications can be used to switch signalling systems, direct molecules to target organelles or terminate biochemical processes [1]. One alternative modification which is much less well studied is sulfation.

**Figure 1.**
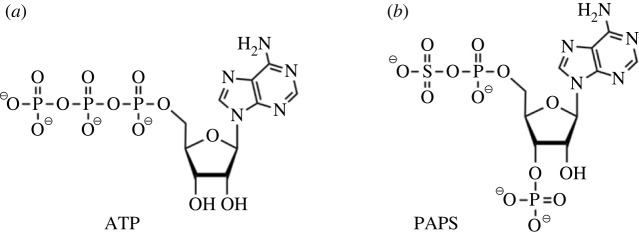
The structures of the phosphate donor ATP (*a*) and the sulfate donor PAPS (*b*).

Phosphorylation and sulfation have evolved to employ the same nucleotide backbone in their respective donors, but these differ in the substitution of the phosphate or sulfate moiety that is to be transferred and their position (figure 1). Furthermore, as nucleic acids appeared first, evolution was able, by re-employing the available molecules, to incorporate sulfate and thereby generate additional diversity. In this way, increasingly complex biological systems could be regulated or modified from the available molecular repertoire without the need for an overwhelming increase in the number of structural genes.

In broad terms, phosphorylation is integral to the structure of nucleic acid polymers, energy production, protein synthesis, intracellular and extracellular signalling (although extracellular polyphosphorylated proteins are relatively rare), while sulfation is linked to extracellular signalling, hormone regulation and cell degradation [2]. The ability to tune and control signalling systems that is provided by phosphates, coupled with their stability in the form of *O*-linked, singly charged, di-esters that join bases in polynucleic acids, may explain their early evolutionary involvement in fundamental mechanisms of reproduction and metabolism [3]. Sulfate mono-esters, on the other hand, occur in a range of roles that are broadly distinct from those to which phosphates are put, including in sulfoglycosphingolipids [4] and as a means of increasing solubility and assisting clearance of dietary and environmental toxins [5]. Comparisons of phosphate and sulfate groups have been made previously, in particular, concerning analysis of their stereochemical properties and protein binding complementarity, structural information having been garnered from X-ray crystal structures [6], as have comparisons of their incorporation, or removal, by enzymes [7]. Here, we survey how the two modifications differ, in regard to both their properties and the biological functions to which they have evolved, and attempt to identify links between them, some of which are only beginning to be explored.

As the most energetically favourable interactions available between molecular species, charge–charge interactions not only allow long-range recognition owing to Coulombic forces (proportional to the inverse square of their distance apart) and relatively high energy binding (proportional to the inverse of their spatial separation and often involving solvent entropic components), but are also subject to the influence of counter ions, especially metal cations; a property which provides further potential means by which the properties of molecules nearing them may be tuned or differentiated. There is a disparity in the extent of the literature concerning these two forms of modification, which has traditionally focused on phosphorylation, although protein sulfation as a post-translational modification of tyrosine residues is increasingly recognized as an important regulator of cell communications and responses [8–13].

Nature employs the addition of charged groups to biological molecules in roles that range from structural components of, most notably, the nucleic acids, to facilitating control of macromolecular interactions and increasing the solubility of toxins to assist their clearance. Phosphorylation emerged early in evolution as the dominant means of introducing charges though the action of single enzymes [14,15] and the conclusion that phosphates cannot be replaced like-for-like by sulfates has been drawn [7].

Here, we assess the properties of these two widespread and seemingly very similar anionic groups in biochemistry as modifications to macromolecules, examine the origins and consequences of their contrasting properties and relate these to their roles. We highlight the biochemical and evolutionary opportunities that their distinct characteristics provided, as well as the advantages offered by the interplay between those signalling systems that exploit them.

## 2. Evolutionary links between phosphorylation and sulfation

Phosphorylation is employed as both the fundamental energy currency and as the principal means of activating intracellular biochemical events and pathways, predominantly using the same molecule, adenosine triphosphate (ATP), as the phosphate donor, although there are limited instances in which pyrophosphate [16–18], guanosine triphosphate (GTP) [19] and adenosine diphosphate (ADP) [20] serve this role. With very few exceptions, for example, bacterial sulfotransferases (STs) that use phenolic sulfates [21] and which show evidence of having evolved in a convergent manner, sulfation employs the sulfate donor 3′-phosphoadenosine-5′-phosphosulfate (PAPS), which is derived using phosphorylation machinery (figure 1).

**Figure 2.**
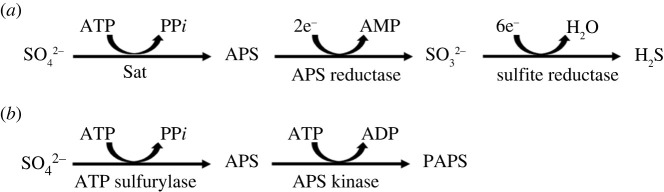
(*a*) Reduction of sulfate (${\mathrm{SO}}_{4}^{2-}$) to sulfite and sulfide (H_2_S) by sulfate reducing bacteria. The process is initiated by the consumption of ATP to form APS, adenosine 5′-phosphosulfate, catalysed by sulfate adenylyl transferase (Sat). (*b*) The sulfate donor PAPS is made in two enzymatic steps from the sulfate anion via APS. These activities are fused into a single enzyme, PAPS synthase.

In the biosynthesis of PAPS, the sulfate anion is adenylated using ATP to form adenosine phosphosulfate (APS) by ATPS and APS is then phosphorylated to form PAPS by APS kinase (figure 2*b*). In eukaryotes, ATPS and APS kinase are fused into a single enzyme, PAPS synthase (PAPSS), of which there are two non-equivalent forms in mammals [22] to help overcome the low catalytic efficiency of the forward reaction of ATPS (*K*_eq_ ∼ 10^−8^) [23].

Although linked at the biosynthetic level, the fact that phosphorylation and sulfation are not interchangeable is consistent both with the observation that the two groups possess distinct fundamental characteristics as well as with the hypothesis that these lie at the origin of their distinct roles.

## 3. Differences between phosphate and sulfate groups

While sulfate and phosphate groups do bear superficial similarity, in terms of, for example, their broad geometry, overall size, shared Lewis basicity and the electronegativity of sulfur and phosphorus atoms, there are two key differences that give rise to important distinctions in properties: namely, the valency of sulfur and phosphorous and, related to this, the charge of their resulting (inorganic) sulfate and phosphate ions, hence also their (organic) mono- and di-esters (table 1). The solubility of salts has been explained by reference to the relationship between lattice energy and enthalpy of hydration, insolubility tending to be most pronounced when the cation and anion are of similar sizes, such as barium and sulfate ions.

**Table 1.** Key properties of phosphorus, sulfur, phosphate and sulfate groups, and their esters.

property	phosphorus	sulfur
electronegativity [24,25]	2.19–2.253	2.48–2.589
covalent (double bond) radius (pm) [26]	107(3)	111(2)
geometry (phosphate and sulfate esters) [27,28]	tetrahedral	tetrahedral
enthalpy of hydration (free anions) (kJ mol^−1^) [29]	2765	1080
valency (of central P or S atom)	5 (3s^2^3p^3^)	6 (3s^2^3p^4^)
mono-esters (and charge)^a^	$R\text{–}O\text{–}{\mathrm{PO}}_{3}^{2-}\;(-2)$	$R\text{–}O\text{–}{\mathrm{SO}}_{3}^{-}\;(-1)$
di-esters (and charge)	$R\text{–}O\text{–}{\mathrm{PO}}_{2}^{-}\text{–}O\text{–}R^{\prime }\;(-1)$	R–O–SO_2_–O–R' (0)
		

^a^The pK_a_ of the sulfate anion is low (1.92), ensuring that anion and ester are negatively charged under all physiological conditions. By contrast, the pK_a_ of phosphate ($H_{2}{\mathrm{PO}}_{4}^{-}$/${\mathrm{HPO}}_{4}^{2-}$) is 7.2, indicating that the anion exists in approximately 1 to 1 ratio of the two forms at pH 7.

Both anions form stable, ordered clusters [30], although the hydration shell of phosphates is larger than for sulfates, each oxygen hydrogen bonding 3 water molecules with strong attraction, providing 13 bound water molecules in the first cell [30], while sulfate binds water more weakly and in smaller rings [31,32].

One consequence is that the solubility of common salts of inorganic phosphate (${\mathrm{PO}}_{4}^{3-}$) and sulfate (${\mathrm{SO}}_{4}^{2-}$) are widely different (table 2). Inorganic phosphate salts of the group II elements calcium and magnesium are considerably less soluble than their corresponding sulfates; the ratio of the solubility of sulfate to phosphate being 132 for calcium and 150 000 for magnesium. Sulfate anions are also more effective than phosphate at causing the ‘salting out' of proteins, according to the Hofmeister series, an effect that has been explained in terms of both electrostatic interactions [33] and solvation energies [34,35].

**Table 2.** Solubility of common phosphate and sulfate salts (g l^−1^ at 20°C) [37].

cation	sulfate	phosphate	ratio (solubility (sulfate/phosphate))
Na	40.8	12.2	3.37
K	13.0	108.0	0.12
Mg	39.7	2.6 × 10^−4^	1.5 × 10^5^
Ca	0.26	0.002 (10°C)	132
Zn	61.3	insoluble	—

A second distinction, however, is that phosphates form both doubly and singly charged phospho- mono- ($R\text{–}O\text{–}{\mathrm{PO}}_{3}^{2-}$) or di-esters (R–O–PO_2_^−^–O–R′), respectively, with organic compounds. By contrast, sulfates form singly charged mono-esters ($R\text{–}O\text{–}{\mathrm{SO}}_{3}^{-}$) with broad cation compatibility, while their di-esters ($R\text{–}O\text{–}{\mathrm{SO}}_{2}\text{–}O\text{–}R^{\prime }$) are uncharged. It has been considered that during early evolution, calcium ions were simply a toxin owing to precisely this propensity to precipitate simple metabolites, particularly phosphates [36]. When attached to biological macromolecules, the mono-ester sulfate groups ($R\text{–}{\mathrm{OSO}}_{3}^{-}$) provide a more versatile means of binding cations than the corresponding doubly charged phosphates ($R\text{–}{\mathrm{OPO}}_{3}^{2-}$) in that, as singly negatively charged ions, they have wider cation compatibility (table 2). This enables sulfated molecules to remain soluble in both calcium and magnesium salt forms, as well as with zinc in low amounts. Thus, the formation of singly charged sulfate mono-esters, rather than doubly charged phosphate mono-esters, provided a means of introducing further charges into biological molecules while avoiding solubility limitations. Furthermore, the single negative charge introduced by sulfation may avoid unwanted additional hydrogen bonding or salt bridges with, for example, Arg residues on the surface of complementary binding surfaces. This analysis reveals that sulfation can introduce charge without exerting pronounced intramolecular or intermolecular effects, compared with phosphate.

There are also important differences in the properties of the respective di-esters of phosphates and sulfates with evolutionary implications. According to conventional thinking concerning early biogenesis, the assembly of nucleic acids into polymers was a key step. This involved forming two covalent bonds through phosphate di-ester ($R-O-{\mathrm{PO}}_{2}^{-}-O-R^{\prime }$) groups, leaving the single remaining negative charge available to interact with cations, retaining solubility, and thereby enabling key features of nucleic acid biochemistry that involve interaction with magnesium and zinc ions [38]. The interacting group, in this case, the phosphinyl moiety ($\text{–}{\mathrm{PO}}_{2}^{-}\text{–}$), possesses sp or intermediate sp–sp^2^ hybridization at oxygen and the lone pair, acting as a Lewis base, coordinates metal ions (Lewis acids) with a preferred *syn* geometry [39]. Comparable interactions would not be available with the analogous (hypothetical) sulfate di-ester (R–O–SO_2_–O–R′), since it is uncharged. The single charge of the phosphate di-ester may also have facilitated attachment to catalytic mineral surfaces, the stereochemical preference for cation binding determining the selection of the mineral surface, thereby generating complex structures destined to be used as biochemical entities [40]. Mineral contact with nucleic acids has also been proposed to play a role in horizontal gene transfer (reviewed in [41]).

An earlier comparison of the interactions of phosphorylated or sulfated macromolecules with proteins revealed that phosphate binding sites in proteins are more highly conserved than those of the comparable sulfate binding sites [42]. This may reflect evolutionarily deeper, more tightly controlled mechanisms involving phosphates and a contrasting degree of relaxed specificity, which is a feature of the interactions between protein networks and the principal polymeric bearer of sulfate (mono-ester ($R\text{–}O\text{–}{\mathrm{SO}}_{3}^{-}$)) groups, the glycosaminoglycan (GAG) class of polysaccharides (reviewed in [43]), whose evolution has been linked to the development of multicellular organisms [44]. The singly charged sulfate mono-esters of GAGs permit a degree of ion selectivity that is perforce based on the appropriate substitution pattern [45] (i.e. the spacing of anionic groups on the GAG polysaccharide) rather than being dictated by the geometry and multiple charges of an individual anionic group. It also seems likely that this property avoids strong binding by solitary sulfate groups thereby enabling the subsequent exchange of bound cations to higher affinity proteins.

## 4. Control of biochemical processes

The emergence of sulfation enabled the development of several capabilities that, excluding the post-translational sulfation of proteins on tyrosine, are largely distinct from those to which phosphorylation has been put. These roles for sulfation include the neutralization of hormones to allow diffusion to their target where they are then de-sulfated to revert to their active form [46], the derivatization of toxic phenolic compounds (e.g. *p*-cresol) [47] from the breakdown of tyrosine, to increase solubility and facilitate excretion, and the diversification of polysaccharide structure to increase the repertoire of potential interactions with proteins [48].

The majority of proteins are regulated by post-translational modification and these also contribute to evolutionary changes. Enzymatic phosphorylation (mono-ester formation) of a free hydroxyl or other analogous functional group, such as an amine, alters protein stability, localization and interactions, and is itself a source of biological diversity. There are two main modes of action, which can be termed either a *switch*, or *aggregate action* [49]. In the first, phosphorylation results in a drastic change of activity (via conformational change or activation of a previously inactive form) to the extent that a two-state system can be considered to have been formed: *on* or *off*. In the second, a more complex situation prevails, in which multiple phosphorylation sites are substituted to varying extents to provide a population containing proteins with a range of activities [50]. Additionally, the combination of phosphorylation sites may dictate a level of activity or, in some cases, the *on* or *off* status. Although based on very different macromolecular scaffolds, the latter situation bears some similarity to the sulfated GAG polysaccharides, in as much as several active forms exist and, among these, there can be varying levels of activity [43]. In eukaryotes, phosphorylation of Ser, Thr and Tyr amino acid side chains is well established while, in prokaryotes, phosphorylation also includes His and Cys derivatives [51,52]. As many as 30% of eukaryotic proteins may be phosphorylated and many of these are phosphorylated at several sites [53,54]. This basic picture has, however, had to be revised following discovery of additional phosphorylated amino acids, including Arg, Lys and Asp [55–58]. It has been suggested that phosphorylation of Ser, Thr and Tyr residues may have evolved from the negatively charged Asp and Glu residues (mutations of Asp or Glu to Tyr require only one G to U substitution) and this could explain why, in some cases, phosphorylation can activate proteins, having effectively evolved from a permanently *on* state to a switchable state, using phosphorylation as the control mechanism [50].

## 5. Sulfation provides an economical route to diversifying signalling capabilities

Since there were, presumably, no biological catalysts or metabolic processes initially, the combination of available chemicals, together with atmospheric and geochemical conditions, are generally held to have been responsible for the generation of the first biological molecules [59]. The ability to use environmental sulfate as a major energy source is also very ancient, having been exploited by sulfate reducing bacteria, which are among the earliest known, dating from about 3.5 Ga and are considered to have played a key role in establishing the sulfur cycle on Earth [60,61]. In this process, sulfur forms a major source of energy, which is highly conserved [62], termed dissimilatory sulfate reduction (DSR; distinct from assimilatory sulfate reduction (ASR) used by many bacteria for the biochemical incorporation of small amounts of sulfur into organic biomolecules). Sulfate anions are transported from the surrounding environment into the cell through a range of sulfate transporters [63], and the key enzyme, sulfate adenylyl transferase (Sat) (ATP sulphurylase), converts sulfate into APS with the consumption of ATP in 1 : 1 stoichiometry (figure 2*a*). This process supplies a terminal electron acceptor for the electron transport chain and then reduces sulfate, first to sulphite, then to hydrogen sulfide, enabling the net production of ATP [64,65]. Of particular note to our discussion, however, is the evolutionarily ancient association of phosphorylation and sulfation, as well as the observation that ATP is required to ‘prime' the DSR pathway.

Prokaryotes possess fully operational signalling systems based on phosphorylation [66,67] and genetic analysis shows that the filistera *Capsaspora owczarzaki* possesses diverse kinases [68], while the tyrosine kinase signalling system probably evolved before divergence of this organism from the common branch containing choanoflagellates and metazoans about 600 Ma. In the sponge *Amphimedia queenslandia*, an early metazoan, there are around 150 receptor tyrosine kinases, such as epidermal growth factor and MET receptors.

To evolve more complex life forms, especially those incorporating multicellular assembly and coordination, it would have been more efficient to expand the number and subtlety of signalling events, than to evolve novel ways of signalling [69]. Such enabling complexity can be achieved to some extent by expanding pathways, combining protein domains and so on, building from the existing molecular machinery and chemical repertoire. It has been estimated that the move from (the presumed) pre-existing levels to the complexity required by choanoflagellates or metazoans involved a significant increase in Src homology 2 (SH2) and protein tyrosine phosphatase (PTP) domains [70–73]. The additional expansion, first evident with the rise of the metazoans [44] and provision of a graded response, both in support of signalling but also its inhibition, was supplied by the advent of the GAG polysaccharides (which, with the sole exception of hyaluronate, are sulfated) and some of which are obligatory co-receptors in cellular signalling. These pathways are modulated through their differential activation and inhibition, a prime example being the fibroblast growth factor/FGFR signalling network (involving tyrosine kinase activity; see also §6), the details of which are beginning to be elucidated in relation to GAG structure and substitution pattern [48,74,75]. Gradients can also be established and maintained through regulation of chemokines and cytokines to influence differential responses (reviewed in [76]). Thus, a direct link exists between extracellular signalling driven by sulfation and intracellular signalling driven by phosphorylation. Sulfated GAGs may thereby serve as expanders and modulators of the existing signalling capability.

The biochemical sulfation of GAGs is achieved through Golgi ST enzymes, which employ PAPS as the sulfate donor, but the origins of these enzymes are subject to discussion. One bioinformatics study [44] identified evidence of homology between 3-*O*-sulfotransferase (3OST) enzymes (HS3ST 1, 2, 3A, 3B, 4, 5 and 6) and sequences in bacteria, but it is not known whether these represent the identification of ancestral proteins that are the originators of other STs in higher organisms. If so, this would be consistent with the observed presence of more 3OSTs than any other form of ST in the GAG biosynthetic machinery of metazoans [77]. Alternatively, they may be examples of convergent evolution although, since HS3STs are related to other heparan sulfate (HS) STs, it is more difficult to see why the relationship is with 3OSTs in particular, and not across the ST family. A further possibility is that these STs could have arisen by horizontal gene transfer from a higher organism, but this raises the question why, seemingly, only 3OST was transferred and whether higher degrees of homology would be expected.

## 6. Interactions between systems using phosphorylation and sulfation merit further investigation

**Figure 3.**
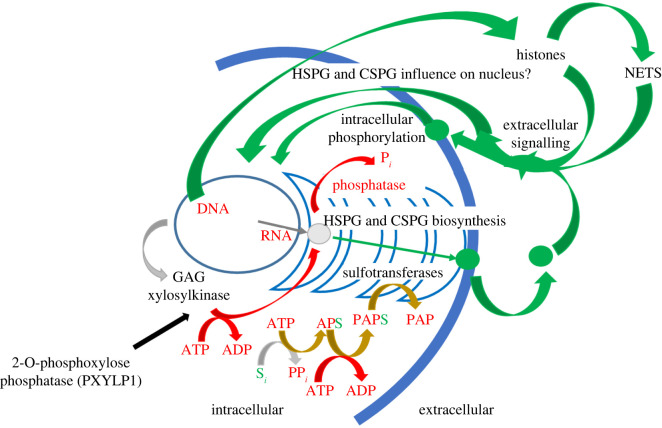
Scheme relating phosphorylation (red) and sulfation (green); the control mechanism is provided by GAG xylosylkinase, which adds a phosphate group to xylose residues linking the nascent GAG to serine of the parent proteoglycan at the onset of GAG biosynthesis in the endoplasmic reticulum (grey), or removal of the 2-O-phosphate group from xylose by PXYLP1 (black). Proteoglycans and their GAG components then interact indirectly via numerous receptors, many of them kinases, eliciting cellular responses intra- and intercellularly, or directly. The overlap of the sulfation and phosphorylation pathway via APS and PAPS is highlighted (brown). The potential influence of events in the nucleus by the sulfated GAGs may complete a possible feedback loop involving both phosphorylation and sulfation.

In addition to sulfated GAG-mediated fibroblast growth factor receptor signalling involving tyrosine phosphorylation (§5), there are further intriguing possibilities of the interaction between systems using phosphorylation and sulfation that have been explored. One is the suggestion that sulfated GAGs can enter the cell nucleus (e.g. [78]) and may be involved in transporting proteins into the nucleus [79]. The hypothesis is that sulfated GAGs may influence processes in the nucleus directly, presumably through interactions with nuclear proteins [80,81]. This is supported by evidence that distinct sulfation patterns in chondroitin sulfate (CS) GAGs regulate the transcription factor Otx2 that mediates neuronal plasticity via upregulation of 6-O-sulfation in CS [82,83]. At the junction of two neurons, Otx2 is released by one neuron and is bound by CS of the adjacent neuron. Following internalization, it is transported along the neuron and then released from the vesicle to act in the nucleus. Such a capability provides a route through which sulfation can influence subsequent phosphorylation events (figure 3). The others relate to the extracellular functions of nuclear, DNA binding proteins and DNA in disease. For example, histones are often released in acute inflammatory conditions, and their level can predict acute organ failure and mortality in acute pancreatitis [84] and, with neutrophil DNA, they are integral to neutrophil extracellular traps (NETs) [85] (figure 3), the formation of which can be induced by heparin [86].

A direct link between phosphorylation and sulfated GAG biosynthesis is, however, provided by Fam20B, which encodes a GAG xylosylkinase [87,88], for the phosphorylation of the xylose sugar residue of the proteoglycan tetrasaccharide linkage region. The phosphorylated xylose stimulates the addition of galactose to the linker region by galactosyltransferase II, increasing its rate of synthesis. While kinases are molecular activators and signal transducers that can regulate cellular processes by adding phosphate groups to diverse target molecules, most of the attention paid to kinase activities has focused on the modification of protein targets in the cytoplasm and nucleus. The biosynthesis of CS or HS type proteoglycans is determined by the identity of the first residue in the chain following this tetrasaccharide linker; GalNAc denoting CS and GlcNAc, the HS type, but phosphorylation of xylose residues results in significantly more efficient elongation by addition of a galactose residue of the GAG tetrasaccharide, a precursor in sulfated GAG chain biosynthesis for a range of proteoglycans [87,88]. Furthermore, the enzyme, 2-O-phosphoxylose phosphatase (PXYLP1) has been shown to catalyse the removal of the phosphate group from the xylose residues of GAGs and influence CS GAG biosynthesis [89,90]. Thus, controlling the phosphorylation status of the nascent chain is emerging as a means of regulating the biosynthesis of sulfated GAG polysaccharides (figure 3).

GAG xylosylkinase, Fam20B, activity and specificity are evolutionarily conserved, and its presence can be traced to the oldest animal phylum. It should be noted, however, that these are the sponges, which do not produce sulfated GAGs. Nonetheless, sponges do possess homologues of XylT, GalT-I, GalT-II and GlcAT and have tetrasaccharide linkers with a phosphorylated xylose residue. These arguments suggest that the appearance of Fam20B xylosylkinase may have set the scene for the biosynthesis of complex sulfated glycan molecules, such as the GAGs, that supported the evolution of complex life and points to phosphorylation as a molecular switch for their appearance.

## 7. Conclusion

While the enzymatic addition of phosphate and sulfate groups bestows negative charges upon their target molecules, their fundamental chemical properties distinguish both the types of linkages that are available to each and the subsequent characteristics of molecules so derivatized. These properties, in turn, dictate the resulting functions for which they have evolved in biological systems and impose a basic dichotomy of roles on the two forms of modification. At the molecular level, examples of interactions between proteins and phosphorylated peptides are provided by studies of SH2 domains [91], and those between sulfated GAGs and proteins by studies of antithrombin [92] and FGFs [93].

Phosphorylation and sulfation are, nevertheless, linked at the biosynthetic level of the formation of the sulfate donor PAPS from APS and ATP (figure 2*b* and figure 3). Phosphorylation also acts as the switch at the initial stages of the biosynthesis of the most heavily sulfated biological macromolecules, the GAG polysaccharides, and these are the carbohydrate component of a major family of proteoglycans. These proteoglycans are involved in regulating numerous protein networks through both intra- and intercellular signalling and include numerous kinases. Their interactions with proteins are characterized by relatively relaxed structural specificity, in contrast to much phosphorylation-driven signalling, several potential GAG sequences being able to interact effectively with a given protein and, often, a given GAG sequence can interact with several proteins, albeit with different binding characteristics.

Exogenously added GAG components detected in the cell nucleus [78] raise the intriguing possibility of interactions with nuclear proteins. Thus, a potential feedback mechanism can be hypothesized which warrants investigation (figure 3). It has long been known that histones, for example, can be isolated through their interactions with immobilized heparin—a sulfated GAG [81]—and extracellularly, this is linked to their activity in acute inflammatory diseases. Similarly, it is likely that the extracellular DNA of NETs interacts with a subset of HS-binding proteins, altering their regulatory functions, though this has not been formally established. It will be interesting to learn what differences exist between the modes of binding of phosphorylated and sulfated molecules—derivatized in these two distinct ways—to those proteins to which they bind selectively, or to which they both bind. Early efforts were made to address this question [42] but, to the best of our knowledge, there has not previously been an extensive comparative study of the properties of protein binding sites for DNA, RNA and GAG-binding proteins.

The repertoire of available biomolecules is relatively limited and the addition of charges via phosphorylation and/or sulfation offers a means of delivering molecular diversity to support correspondingly more elaborate signalling events during the evolution of increasingly complex life forms. Ultimately, by employing already existing biochemical apparatus to make these two distinct derivatizations, biological systems have been able to expand their repertoire of modification and, hence, ways of signalling, efficiently. The points at which these occurred during evolution, the full extent of the interactions between the two systems, and their consequences on the subsequent development of advanced complex life forms are all topics worthy of further exploration.

## Data Availability

No experimental data have been included in this review.
